# Effect of denosumab on glucose metabolism in postmenopausal osteoporotic women with prediabetes: a study protocol for a 12-month multicenter, open-label, randomized controlled trial

**DOI:** 10.1186/s13063-023-07769-0

**Published:** 2023-12-18

**Authors:** Yilin Wang, Yu Jiang, Jia Li, Xisheng Lin, Yan Luo, Shuhuai Tan, Haohan Yang, Zefu Gao, Xiang Cui, Pengbin Yin, Dan Kong, Yuan Gao, Yu Cheng, Licheng Zhang, Peifu Tang, Houchen Lyu

**Affiliations:** 1 Medical School of Chinese PLA, Beijing, 100853 China; 2 Department of Orthopedics, Chinese PLA General Hospital, No. 28, Fuxing Road, Beijing, 100853 People’s Republic of China; 3 National Clinical Research Center for Orthopedics, Sports Medicine & Rehabilitation, Beijing, 100853 China; 4 Department of Rehabilitation, the Second Medical Center of Chinese PLA General Hospital, Beijing, 100853 China; 5 Department of Nursing, the First Medical Center of Chinese PLA General Hospital, Beijing, 100853 China; 6 Department of Endocrinology, Chinese PLA General Hospital, Beijing, 100853 China

**Keywords:** Denosumab, Glucose metabolism, Osteoporosis, Postmenopausal women, Prediabetes, RANKL

## Abstract

**Background:**

Participants with prediabetes are at a high risk of developing type 2 diabetes (T2D). Recent studies have suggested that blocking the receptor activator of nuclear factor-κB ligand (RANKL) may improve glucose metabolism and delay the development of T2D. However, the effect of denosumab, a fully human monoclonal antibody that inhibits RANKL, on glycemic parameters in the prediabetes population is uncertain. We aim to examine the effect of denosumab on glucose metabolism in postmenopausal women with osteoporosis and prediabetes.

**Methods:**

This is a 12-month multicenter, open-label, randomized controlled trial involving postmenopausal women who have been diagnosed with both osteoporosis and prediabetes. Osteoporosis is defined by the World Health Organization (WHO) as a bone mineral density T score of ≤ − 2.5, as measured by dual-energy X-ray absorptiometry (DXA). Prediabetes is defined as (i) a fasting plasma glucose level of 100–125 mg/dL, (ii) a 2-hour plasma glucose level of 140–199 mg/dL, or (iii) a glycosylated hemoglobin A1c (HbA1c) level of 5.7–6.4%. A total of 346 eligible subjects will be randomly assigned in a 1:1 ratio to receive either subcutaneous denosumab 60 mg every 6 months or oral alendronate 70 mg every week for 12 months. The primary outcome is the change in HbA1c levels from baseline to 12 months. Secondary outcomes include changes in fasting and 2-hour blood glucose levels, serum insulin levels, C-peptide levels, and insulin sensitivity from baseline to 12 months, and the incidence of T2D at the end of the study. Follow-up visits will be scheduled at 3, 6, 9, and 12 months.

**Discussion:**

This study aims to provide evidence on the efficacy of denosumab on glucose metabolism in postmenopausal women with osteoporosis and prediabetes. The results derived from this clinical trial may provide insight into the potential of denosumab in preventing T2D in high-risk populations.

**Trial registration:**

This study had been registered in the Chinese Clinical Trials Registry. Registration number: ChiCTR2300070789 on April 23, 2023. https://www.chictr.org.cn.

**Supplementary Information:**

The online version contains supplementary material available at 10.1186/s13063-023-07769-0.

## Background

Prediabetes is strongly correlated with type 2 diabetes (T2D) [1]. According to the International Diabetes Federation, the estimated number of adults worldwide with impaired glucose tolerance or impaired fasting glucose in 2021 is 541 million and 319 million, respectively [2]. Annually, 5–10% of individuals with prediabetes develop T2D, while the life-term risk is placed at 70% [3]. Despite the availability of multiple classes of antidiabetic medications, improving glucose management in the prediabetic population remains an unmet medical need, and new pharmacological treatments are warranted [4, 5].

Compelling epidemiological and experimental evidence suggests that the receptor activator of nuclear factor-κB ligand (RANKL) plays a pivotal role in the pathogenesis of T2D. In a large prospective population-based study, a high level of serum soluble RANKL has been identified as an independent risk factor of T2D in the general population [6]. Individuals with higher levels of RANKL are at a higher risk of T2D (middle versus low group: odds ratio (OR), 3.37; 95% CI, 1.63–6.97; high versus low group: OR, 4.06; 95% CI, 2.01–8.20) [7]. On the other hand, blockade of the RANKL signaling pathway can improve hepatic insulin sensitivity, induce pancreatic β-cell proliferation, and ameliorate or even normalize hyperglycemia in a diabetic murine model [7, 8]. Therefore, the reduction of RANKL activity may present a viable approach to the prevention or delay of T2D.

Denosumab, a fully human monoclonal antibody that effectively inhibits RANKL, has been widely used for the management of osteoporosis [9]. However, the effect of denosumab on glycemic parameters is still under investigation, with mixed results from various studies [10–16]. Both observational studies and post hoc analyses of randomized controlled trials (RCTs) have been conducted, but most of them were small and prone to confounding. The heterogeneity of the study population, including different risks of developing type 2 diabetes (non-diabetes, prediabetes, or diabetes), could dilute the effect of denosumab on glycemic parameters. Furthermore, no RCT with adequate power on glycemic parameters in a prediabetes population has been reported yet.

Despite the inconclusive findings, some studies have suggested that denosumab may have a positive effect on glycemic metabolism [10–16]. A retrospective study of subjects with prediabetes or T2D showed that denosumab significantly reduced glycosylated hemoglobin A1c (HbA1c) and fasting plasma glucose (FPG) after 12 months, compared to calcium and vitamin D [10]. However, the post hoc analysis of the FREEDOM (Fracture REduction Evaluation of Denosumab in Osteoporosis Every 6 Months) study did not show improvements in glycemic parameters in the total population. Instead, improvements were observed only in a subgroup of women with T2D not receiving anti-diabetic medication [15, 16]. A recent study in adults with osteoporosis found that the use of denosumab was associated with a lower risk of developing type 2 diabetes compared to the use of oral bisphosphonates [17]. Specifically, the initiation of denosumab reduced the risk of incident T2D by a hazard ratio of 0.68 (95% CI 0.52 to 0.89). Individuals with a higher risk of T2D – those with prediabetes or obesity – appeared to derive greater benefit from denosumab initiation than from oral bisphosphonate use with hazard ratios of 0.54 (95% Cl 0.35 to 0.82) and 0.65 (95% Cl 0.40 to 1.06), respectively. These findings indicate the importance of RCTs in further evaluating the potential benefits of denosumab in reducing the risk of T2D in individuals with osteoporosis.

In this study, we plan to perform a 12-month multicenter, open-label, parallel-group, superiority RCT, examining the effect of denosumab on the level of HbA1c at 12 months in postmenopausal women with osteoporosis and prediabetes. An RCT with adequate power in a prediabetes population could help clarify the effect of denosumab on glycemic parameters and provide more conclusive evidence to guide decision-making.

## Methods

### Trial design

**Fig. 1:**
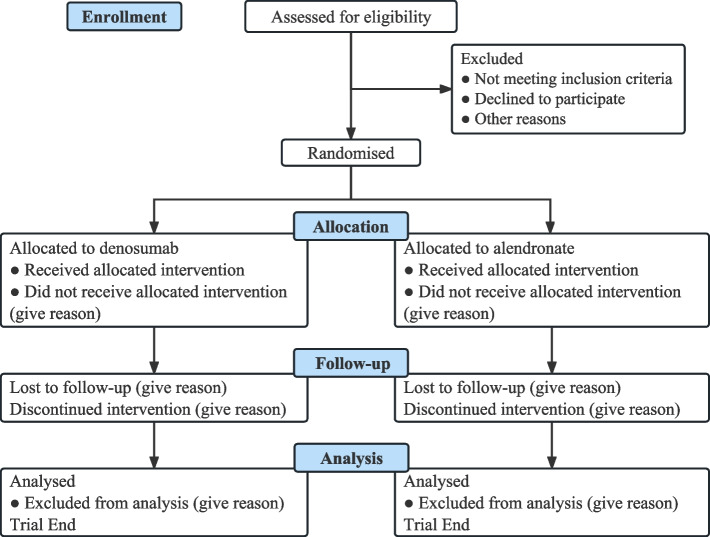
Flow diagram

**Fig. 2:**
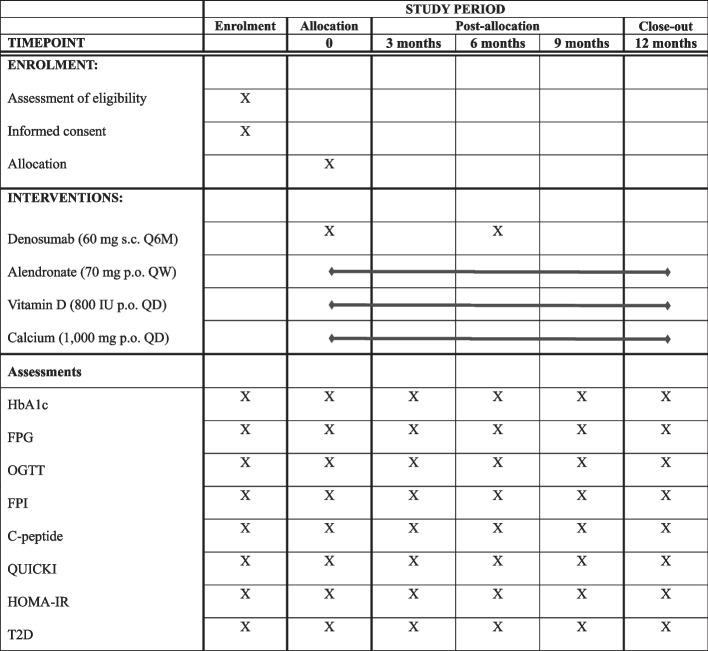
SPIRIT Figure. Abbreviations: s.c., subcutaneous injection; p.o., oral administration; HbA1c, glycosylated hemoglobin A1c; FPG, fasting plasma glucose; OGTT, 2-hour glucose oral glucose tolerance test; FPI, fasting plasma insulin; QUICKI, quantitative insulin-sensitivity check index; HOMA-IR, homeostasis model assessment of insulin resistance; T2D, type 2 diabetes

This is a 12-month multicenter, open-label, parallel-group RCT. Participants in the intervention group will receive denosumab (60 mg s.c. every 6 months), while those in the control group will receive alendronate (70 mg p.o. every week), which is the first-line therapy for patients with postmenopausal osteoporosis [18]. This study protocol has been developed following the recommendations of SPIRIT (Standard Protocol Items: Recommendations for Interventional Trials) 2013 Statement, a guideline for a clinical trial protocol [19]. The SPIRIT checklist and flow diagram of this study protocol are shown in Additional file 1 and Fig. 1, respectively. The schedule for recruitment, intervention, assessment, and visit is shown in Fig. 2.

### Sample size

The sample size was estimated based on the primary outcome, the change in HbA1c from baseline to 12 months. According to a previous study, sample size calculations were based on a projected between-group difference in HbA1c of 0.301% with a standard deviation (SD) of 0.909%. Considering a type I error of 0.05, a study power of 80%, and a 20% drop-out rate, the total sample size was calculated to be 346 (173 per group) using R 4.2.0.

### Setting and participants

The study will recruit a sample size of 346 eligible postmenopausal women with osteoporosis and prediabetes from eight medical centers affiliated with the Chinese People’s Liberation Army General Hospital, Beijing, China.

### Inclusion criteria

Participants to be included in the study must be postmenopausal women with osteoporosis and prediabetes aged 60–90 years. Postmenopausal refers to participants aged 60 or above who had no menses for 12 or more consecutive months. Osteoporosis is defined as a bone mineral density (BMD) T score of − 2.5 or lower at the lumbar spine or total hip by dual-energy X-ray absorptiometry (DXA). Prediabetes is defined as either FPG 100–125 mg/dL, a 2-hour plasma glucose 140–199 mg/dL, or an HbA1c 5.7–6.4%, according to the American Diabetes Association 2010 criteria [20].

### Exclusion criteria

Exclusion criteria include: (i) having T2D or receiving any anti-diabetes medications within 8 weeks before screening; (ii) receiving any anti-osteoporosis medications prior to the screening process; (iii) receiving any postmenopausal hormone therapy; (iv) presence of an active infection (e.g., HIV, active tuberculosis) or severe illness (e.g., severe cardiovascular disease, cancer, endocrine disorders); (v) severe allergy to denosumab or any component of the formulation; (vi) uncontrolled hypocalcemia; (vii) blood donation or blood loss of > 400 ml (mL) within the last year; (viii) planned invasive dental surgery within the next year (e.g., tooth extraction, dental implants, oral surgery).

### Intervention

Eligible participants will be randomly assigned in a 1:1 ratio to receive either subcutaneous denosumab 60 mg every 6 months or oral alendronate 70 mg every week. Alendronate is the first-line treatment for osteoporosis. All participants will receive oral calcium 1000 mg/d and vitamin D 800 IU/d. We will evaluate participants’ adherence to interventions using verbal interviews and counting the remaining tablets during each follow-up visit.

All participants will receive the same monthly lifestyle counseling throughout the study. They will be advised to engage in sufficient physical activity (at least 150 minutes per week) and to reduce their daily calorie intake by at least 500 kcal below their daily energy requirement. Baseline dietary and exercise advice will be provided by a registered dietitian through a face-to-face interview (30 minutes). The compliance to the intervention will be evaluated during the last follow-up visit. Self-rated assessments will be used to quantify participants’ physical activity (0 = “low exercise volume (almost no exercise)”, 1 = “medium exercise (< 150 minutes per week)”, and 2 = “high exercise volume (≥ 150 minutes per week)”) and the extent of calorie intake reduction (0 = “≤ 10%”, 1 = “10–25%”, and 2 = “25–50%”, 3 = “≥ 50%”). We will conduct a sensitivity analysis by adjusting for the compliance of physical activity and calorie intake in the multiple regression models.

### Discontinuation criteria

Participant discontinuation from the study may occur due to any of the following reasons: (i) loss to follow-up or withdrawal of consent; (ii) incidence of serious adverse events (SAEs) such as atypical femur fracture, severe hypoglycemia, serious infection, osteonecrosis of the jaw, and death; (iii) concurrent use of any antidiabetic medication (including insulin, oral antidiabetic drugs, or Traditional Chinese Herbal medicine); (iv) use of alternative anti-osteoporotic agents other than denosumab or alendronate. Post-randomization, any participant diagnosed with type 2 diabetes or who begins taking any antidiabetic medication will be censored and referred to clinicians for consultation regarding standard hypoglycemic treatments.

### Study outcomes

The primary outcome is the change in HbA1c from baseline at 12 months. Secondary outcomes include: (i) changes in FPG and 2-hour plasma glucose from baseline to 12 months; (ii) incidence of T2D, diagnosed by either FPG ≥ 126 mg/dL, or 2-hour plasma glucose ≥200 mg/dL, or HbA1c ≥ 6.5%, or self-reported diagnosis of diabetes (regardless of antidiabetic medication use), or use of antidiabetic medication [20]; (iii) changes in FPI and C-peptide from baseline to 12 months; (iv) changes in insulin sensitivity and insulin resistance from baseline to 12 months. Insulin sensitivity and insulin resistance will be determined using the quantitative insulin-sensitivity check index (QUICKI) and the homeostatic model assessment of insulin resistance (HOMA-IR) equation, respectively [21, 22].

$$\textrm{QUICKI}=1/\left\{\ \right[\log\ \left[\textrm{FPI}\ \left(\upmu \textrm{U}/\textrm{mL}\right)\right]+\log\ \left[\textrm{FPI}\ \left(\textrm{mg}/\textrm{dL}\right)\right]\Big\}$$

$$\textrm{HOMA}-\textrm{IR}=\textrm{FPI}\ \left(\textrm{mU}/\textrm{L}\right)\ast \textrm{FPG}\ \left(\textrm{mmol}/\textrm{L}\right)/22.5$$

### Recruitment

We will employ an offline approach to screen and recruit study participants at the clinics. However, considering the limited awareness of osteoporosis in those with prediabetes, we plan to increase awareness by utilizing media advertising. This study was approved to recruit participants from eight medical centers from Oct 2023. The study has received institutional ethics approval. Written informed consent will be obtained from all participants prior to the screening phase. Consecutive participants who fulfill the inclusion criteria and are willing to participate will be considered.

### Randomization and blinding

The ResMan web response system (http://www.medresman.org/login.aspx) will be implemented for the random assignments of study participants in a 1:1 ratio to either the denosumab or alendronate group. To ensure balanced distribution, randomization will be stratified by center. While participants and doctors will not be blinded to the treatment assignment, those involved in assessing the outcomes will be blinded to the information. A procedure for unblinding is not necessary.

### Safety assessment

Each participant will be assessed for any adverse events (AEs) during the clinical study. The nature of the AEs, severity, duration, treatment, and outcome will be documented and analyzed to determine if they are related to the study drug.

### Data collection and management

All pertinent information for each subject will be documented in the paper case report form (CRF) and entered into the ResMan in a timely and accurate manner by trained study personnel. Standard data coding has been predefined by researchers for all raw, non-numeric data. Randomization will be stratified by center using the ResMan system. To enhance data quality, the study personnel will employ several strategies, including double data entry and range checks for data values during study analyses. Figure 2 provides an overview of the data collection schedule. Following enrollment, participants will undergo evaluations every 3 months for a duration of 12 months using the same data collection methods as utilized during the baseline visit. Blood tests will be conducted at the Department of Clinical Laboratory at each visit, and quality control procedures and calibration will be performed in accordance with the International Standardization Organization (ISO) 15,189. Data will be securely stored in the ResMan database system, utilizing password-protection measures. All information will be preserved independently as double copies, minimizing the risk of data loss and enabling data backup.

### Data monitoring

The Data Monitoring Committee from the Department of Orthopedics of PLA General Hospital will oversee the quality of the data and ensure the safety of the participants. The National Clinical Research Center for Orthopedics, Sports Medicine, and Rehabilitation will conduct an audit of this study every 6 months.

### Harms

We will use the Common Terminology Criteria for Adverse Events (CTCAE Version 5.0) to evaluate the AEs in this study [23]. Investigators will interview participants for any potential AEs and SAEs at every visit.

A total of approximately 100 mL of blood will be drawn during the blood test for HbA1c, FPG, 2-hour plasma glucose, FPI, and C-peptide throughout the 12-month follow-up period. This exercise is not expected to cause any unusual discomfort to the study subjects.

Bone density measurements will be tested using DXA. The total dose of radiation during the DXA scan (about 2.5 μSv) is significantly lower than the current recommendations for regulatory annual dose limits for radiation workers and members of the public, which are 20 mSv and 1 mSv, respectively [24].

Concerns related to AEs of denosumab include hypocalcemia, osteonecrosis of the jaw, atypical femur fractures, and serious infection. It is recommended that denosumab should not be given to participants with pre-existing hypocalcemia until it is corrected. Literature reports suggest that the risk of SAEs with the use of denosumab is low [25]. It has been observed that discontinuing or delaying denosumab results in bone loss and increases the risk of vertebral fractures within a relatively short period of time [26–28]. During the study, if participants withdraw from the denosumab arm, we will recommend that they either continue denosumab or switch to bisphosphonate therapy for long-term management of osteoporosis [29].

### Provisions for post-trial care

Participants will be provided with an individualized schedule of assessment appointments and will receive reminders through telephone calls and emails. Participants may meet the predefined criteria for withdrawal at any time during the study period. In the event of an adverse event, completion of the study treatment, or early withdrawal, the treating clinician will provide the necessary routine clinical care if required.

Participants who choose to discontinue treatment are encouraged to attend visits whenever feasible, unless they decide to withdraw their consent. All safety investigations will continue to be conducted according to the investigational schedule until the completion of the study.

### Statistical analysis

Descriptive statistics will be used to summarize the baseline characteristics of the study participants, including age (continuous), treatment group (0, alendronate; 1, denosumab), BMI (continuous), energy intake (continuous), the time since the final menstrual period (FMP) (continuous), and baseline HbA1c, FPG, and OGTT (all continuous). Baseline descriptive data will be compared between the control and intervention groups using the chi-square test for categorical variables and the t-test for continuous variables. The Kolmogorov-Smirnov test will be used to assess normality of each continuous variable. Categorical variables will be presented as frequency (percentage), while continuous variables will be presented as mean (SD) or median (interquartile range) as appropriate.

The analysis of primary and secondary outcomes will be conducted based on the intention-to-treat principle, including all individuals who were randomized and received at least one outcome assessment at baseline. Drug efficacy will be evaluated using mixed linear regression models, accounting for the study center and baseline HbA1c value, to assess group differences and time-by-treatment interactions for repeated outcomes (i.e., the changes in HbA1c, FPG, 2-hour plasma glucose, FPI, and C-peptide) [30, 31]. The fixed effects in the models will include treatment, time indicator (3, 6, 9, 12 months), and possible interactions between treatment and time indicators, while random effects will include participant and study center. We will use Cox regression to compare the incidence of T2D at 12 months between treatment groups. The safety dataset will include all randomized individuals. Safety analyses will include the incidence, severity and type of AEs, and changes in the vital signs and laboratory results. Participants with missing baseline HbA1c values will be excluded from the analysis, while missing values at baseline for other variables will be handled using multiple imputations. Only participants who have at least one follow-up visit will be included in the efficacy analysis. Any missing HbA1c values during the follow-up will be addressed by the last observation carried forward (LOCF) method [32].

We will perform two sensitivity analyses to assess the robustness of the findings. First, a sensitivity analysis will be performed based on the per-protocol principle. Second, we will exclude study participants with missing values of HbA1c during the follow-up.

All statistical analyses will be performed using R version 4.2.0 (R Foundation for Statistical Computing, Vienna, Austria), and a value of *P* < 0.05 will be considered statistically significant.

### Interim analysis

No interim analysis is planned in this study.

### Informed consent form

The informed consent will be reviewed and approved by the institutional review board before the study commences. Researchers will inform participants of the relevant information in a language that is clearly understandable. The participants and their representatives will be given adequate time to read the form and address any research-related questions. The written informed consent of each participant will then be sought and kept strictly confidential. Identifying details will be coded to ensure the anonymity of the participants. No additional consent is obtained because no ancillary studies or studies involving biological specimens are planned.

### Ethics and dissemination

This study will be conducted in adherence to ethical principles outlined in the Declaration of Helsinki [33] and the Good Clinical Practice guidelines [34]. This study was approved by the Ethics Committee of the Chinese PLA General Hospital with the approval number: S2023–059-01. Any changes to the trial protocol will be communicated to the appropriate regulatory authorities and trial participants. If necessary, participants will be asked to provide their consent again. This study is an investigator-initiated RCT, and the principal investigator declares no conflicts of interest. After all participants have completed their visits, all data will be anonymized and maintained by the Chinese PLA General Hospital. No other organization or individual will have access to the data except when required by law. All results will be published in international peer-reviewed journals as a means of disseminating them to the wider scientific community.

## Discussion

This study will provide evidence of the efficacy of denosumab in improving glucose metabolism in postmenopausal women with osteoporosis and prediabetes. Results from this trial are expected to provide insights into the potential role of denosumab in preventing T2D in high-risk populations and may enhance the co-management of prediabetes and osteoporosis.

The prevalence of prediabetes is high among participants with osteoporosis. The FREEDOM trial showed that among the 7808 postmenopausal women with osteoporosis, 16.2% had prediabetes, and 8.5% had diabetes [16]. This association may lie beyond simple coincidence solely due to the high prevalence of two single diseases, as a shared pathogenetic mechanism between T2D and osteoporosis may exist [35]. The RANKL signaling pathway may link bone remodeling to glucose metabolism and provide a new approach for improving glycemic control with anti-RANKL therapies in postmenopausal osteoporotic women with prediabetes. Although previous trials have provided some supporting evidence, there is still a lack of high-quality evidence of denosumab on glucose metabolism, especially in the high-risk population of postmenopausal osteoporotic women with prediabetes. The objective of this study is to examine the potential of denosumab in delaying or preventing the progression of prediabetes to T2D in individuals undergoing osteoporosis treatment. The results of this study may contribute to the evidence of the potential benefits of denosumab on glucose metabolism in this specific subpopulation and shed light on the selection of anti-osteoporotic drugs.

This planned study has several strengths. First, to the best of our knowledge, this will be the first RCT with sufficient power to determine the effect of denosumab on glucose metabolism in postmenopausal osteoporotic women with prediabetes. Second, the study population is well characterized, and the multicenter study design can provide statistical robustness for the target population in different regions, thereby better representing the overall study population. Third, this study will involve a reasonably long follow-up period of 12 months, giving sufficient time to evaluate the hypoglycemic effect of denosumab. Fourth, a comprehensive recruitment strategy was used to ensure efficient recruitment. This included utilizing social media advertisements and routine outpatient services, which facilitates the recruitment of study participants within a relatively short period.

However, some notable limitations are anticipated with this study as well. First, we will only focus on postmenopausal osteoporotic women with prediabetes; osteoporotic men with prediabetes will not be examined. Second, no stratified randomization is performed on other high-risk factors for T2D. However, we will conduct ad-hoc exploratory subgroup analyses. Third, this study may not have sufficient power to adequately analyze secondary endpoints such as FPI, C-peptide, insulin sensitivity, insulin resistance, and the incidence of T2D.

In conclusion, this study will determine the additional benefits of denosumab on glucose metabolism and the development of T2D. It will provide valuable insights into the effects of denosumab on glycemic parameters and have important implications for tailoring individualized drug management of osteoporosis.

### Trial status

The date of registration was 23 April 2023 (protocol version: 1.0 on 1 November 2022). The recruitment began on 1 Oct 2023, and we plan to complete the recruitment on 1 May 2024.

### Supplementary Information

**Additional file 1.** SPIRIT 2013 Checklist: Recommended items to address in a clinical trial protocol and related documents*

**Additional file 2.** Informed consent

## Data Availability

Access to the final trial dataset in this research is available by reasonable request to the corresponding author.

## Abbreviations

AEs: Adverse events
BMD: Bone mineral density
CRF: Case report form
CTCAE: Common Terminology Criteria for Adverse Events
DXA: Dual-energy X-ray absorptiometry
FREEDOM: Fracture REduction Evaluation of Denosumab in Osteoporosis Every 6 Months
FPG: Fasting plasma glucose
HbA1c: Hemoglobin A1c
HOMA-IR: the homeostatic model assessment of insulin resistance
ISO: the International Standardization Organization
mL: milliliter
OR: Odds ratio
RANKL: Receptor activator of nuclear factor-κB ligand
RCT: Randomized controlled trial
SAEs: Serious adverse events
SD: Standard deviation
SPIRIT: Standard Protocol Items: Recommendations for Interventional Trials
T2D: Type 2 diabetes
QUICKI: Quantitative insulin-sensitivity check index
