# Supplementary material for: Effect of denosumab on glucose metabolism in postmenopausal osteoporotic women with prediabetes: a study protocol for a 12-month multicenter, open-label, randomized controlled trial
Source: Trials. 2023 Dec 18;24:812. doi: 10.1186/s13063-023-07769-0 (PMC10726555; doi:10.1186/s13063-023-07769-0)
Supplement: Supplementary file 2 — Additional file 2. Informed consent [file 13063_2023_7769_MOESM2_ESM.docx]

**知情同意书**

# 版本号 v1.0 版本日期:2022.11.01

**尊敬的受试者：** 您好！

我们诚邀您参加一项中国人民解放军总医院第一医学中心骨科批准开展的地舒单抗对合并糖 尿病前期的绝经后骨质疏松女性糖代谢影响的研究。请您用一定的时间仔细阅读下面的内容，如 有不清楚的问题或术语，可以与有关医生进行讨论。

您参加本项研究完全自愿，本研究已经得到中国人民解放军总医院伦理委员会的审查和批准。

# 研究类型：

多中心、开放标签、随机对照试验。

# 研究背景：

糖尿病前期是2型糖尿病(T2DM)的重要危险因素。核因子-κB配体受体激活因子(RANKL)在 T2DM发病中起关键作用。地舒单抗(denosumab)是一种RANKL单克隆抗体，用于治疗骨质疏松症， 临床前研究提示地舒单抗可能有改善糖代谢、降低血糖、阻止糖尿病前期进展为糖尿病的潜力。 然而，地舒单抗对改善糖代谢的具体疗效仍不明确。因此，我们拟通过开展临床随机对照试验， 明确地舒单抗改善合并糖尿病前期的绝经后骨质疏松女性糖代谢的疗效。

# 研究目的：

明确地舒单抗改善合并糖尿病前期的绝经后骨质疏松女性糖代谢的疗效。

# 研究内容：

如果您同意参与这项研究，我们将在您入院时采集您的相关病史信息并建立健康档案，测量 骨密度，检验血糖水平，并加入生物样本库用于后续研究，记录并观察使用抗骨质疏松药物后血 糖变化情况。本研究将招募54名受试者随机分配治疗方案，您将有1/2的机会接受地舒单抗60mg， 皮下注射，1次/半年，共2次；1/2的机会接受阿仑膦酸钠70mg，口服，1次/周，为期1年。

# 研究程序：

本研究持续 12 月，共 4 次随访（用药后 3 月，6 月，9 月，12 月），在此期间，每次随访时 刻，需要到解放军总医院第一医学中心骨科专病门诊或北京地区三甲医院检测血糖、75g口服糖 耐量试验，检验结果及临床不良反应等信息需要电话告知研究者。在整个研究过程中，我们将 通过长期术后随访了解您的血糖情况，并根据病情为您提供相应的指导意见。

**该研究可能会带来的影响：**

本研究属于随机对照试验，两组患者均使用抗骨质疏松药物，不会增加骨质流失、血糖升高额外风险。仍需告知您正常诊疗的相关风险，抗骨质疏松药物的常见不良反应，如胃肠道反应， 急性流感样反应等，但此类风险不属于本研究所致风险。

在整个研究期间您不能再参加其他任何有关药物或者医疗器械的临床研究。

**研究的风险和不良反应：**

研究过程中您可能会出现不良反应。我们会监测研究中所有病人的任何不良反应。如果您在访视之间出现任何不良反应，请及时给您的研究医生打电话咨询。 已知风险：目前，应用地舒单抗最常见引起的不良反应包括：严重低钙血症、过敏反应等。 未知风险：可能存在一些目前无法预知的风险及不良反应。 为此将我们采取以下措施以降低试验的安全性风险：

1.排除具有低钙血症高风险的重度肾功能损害的患者（肌酐清除率＜30ml/min）或接受透析机长 期大量使用糖皮质激素的患者；

2.对于所有受试者给予足够的钙和维生素D补充，用药前检验血钙水平；

3.加强对受试者的宣教，试验过程中有任何不适及时报告医护人员；

4.对医护人员进行针对性培训，密切观察受试者有无过敏相关症状、体征，及时对症处理；

5.试验过程中出现任何其他意外情况，及时报告主要研究者并进行及时、妥善处理。

您需告诉您的家人或与您亲近的朋友您正在参加一项临床研究，他们可以注意上面描述的事

件。如果他们对您参加研究有疑问，您可以告诉他们怎样联系您的研究医生。

# 研究获益：

直接受益：通过对骨密度、血糖检查，有助于临床医生对您的骨骼健康和血糖情况进行更全面 的评估，使用抗骨质疏松药物后，可使骨质疏松症得到改善，后续随访检查也有利于对血糖情况 进行监测。

潜在受益：本研究将确定地舒单抗对糖代谢的影响，从而在治疗骨质疏松症的同时，预防2 型糖尿病的发展，减少2型糖尿病相关并发症。本研究结果可为糖尿病前期骨质疏松患者选择最合适的药物提供重要参考。

**生物标本和医疗信息的处理和利用：**此研究预计不留存生物标本。

**您的权利和义务：**

您有充分的时间考虑和随时提问的权利，且是否要参加本研究的最终决定权在您。如果您决

定不参加本研究，也不会影响您应该得到的其他医学关注；如果您决定参加，请您如实的告诉研 究医生有关自身病史和身体状况的真实情况，告诉研究医生自己是否曾参与其他研究，或目前正 参与其他研究，并请您在这份书面知情同意书上签字。签字后，您仍然可以在研究的任何阶段退 出本研究。如果在研究期间发现任何新的、重要的，并且可能会影响您继续参加这一研究意愿的 信息，您的研究医生或其他研究小组成员会立即通知您。您也可以随时了解和咨询研究情况。如 果您没有遵守研究计划，或者研究医生认为您继续参加本研究不符合您的最大利益，研究医生可 以让您退出研究；如果您出现对研究药物的不良反应，或研究期间有关于研究药物的新的安全性 的信息出现，研究医生或申办者可能会在未征得您同意的情况下终止您参与本项研究。如果您因 为某些原因从研究中退出，您可能被询问有关您使用研究药物的情况。如果研究医生认为需要， 您也可能被要求进行计划外的体格检查和实验室检查，研究医生将会和您讨论退出研究后的医疗 事宜。

**参加研究的相关费用：**

您确定参加本研究后，在药物干预当天早晨，行骨密度检查、空腹血糖检测、空腹胰岛素检

测、C肽浓度检测、75g口服葡萄糖耐量试验。如果被分配至地舒单抗干预组，须加做血钙检验， 以上检查及检验结果均为免费。地舒单抗或阿仑膦酸钠药物免费。

在3、6、9、12月随访时刻，需于解放军总医院骨科专病门诊或北京地区三甲医院就诊，行空 腹血糖检测、空腹胰岛素检测、C肽浓度检测、75g口服葡萄糖耐量试验，以上检验均为免费。

# 报酬或补偿：

本临床研究是在患者本人及家属充分了解相关信息后自愿参加，根据相关伦理法规规定，为 保障临床试验的真实性和有效性，患者不会因参加本研究而获得任何额外报酬或补偿。

**研究所致损害的处理措施：**

如果您的健康确因参加这项研究而发生与研究相关的损害，请立即通知研究医生，研究医生

将负责对您采取适当的治疗措施。您可以获得免费治疗或根据中国法律获得相应的补偿。即使您 已经签署这份知情同意书，您仍然保留您所有的合法权利。如您的权益受到侵犯，您可以联系解 放军总医院医学伦理委员会，电话：010-66937166。

# 保密性：

如果您决定参加本项研究，您参加试验个人信息均严格保密。您的医疗记录将保存在医院， 研究者、研究主管部门、伦理委员会将被允许查阅您的医疗记录。我们将在法律允许的范围内， 尽一切努力保护您个人医疗资料的隐私。

# 自愿参加：

参加本研究是完全自愿的，您可以拒绝参加研究，或者在加入研究过程中的任何时候选择退 出本次研究。该决定不会影响您未来的治疗。研究过程中您可随时了解与本研究有关的信息资料 和研究进展，如果您有与本研究有关的问题，或您在研究过程中发生了任何不适或损伤，或有关 于本项研究参加者权益方面的问题，您可以与研究者联系。

**研究中如何获得帮助：**

您可随时了解与本研究有关的信息资料和研究进展，如果您有与本研究有关的问题，请联系吕医生（13501149301）与王医生（13241690319）联系。 如果您在研究过程需要了解关于本项 研究受试者权益方面的问题您可以联系解放军总医院医学伦理委员会，联系电话 010-66937166。

# 知情同意签字页

**受试者声明：**

我在充分了解该项研究的知情同意书的全部内容以及参加本研究可能带来的风险和受 益后，自愿参加本试验，并做出以下申明：

1. 我已阅读了上述知情同意书中的内容并理解本研究的性质、目的及该项研究可能出现 的不良反应等信息，我的问题已经得到满意的答复。

2. 我将遵守知情同意书中要求，并与研究人员充分合作，如实、客观地向研究人员提供 参加本研究前、研究期间和各随访期的健康状况及相关情况。

3. 我明白我可以随时退出研究，而此后的治疗并不会因此受到任何不利影响。我理解研 究者有权根据我的情况随时终止研究。 4.我知晓我会收到一份签署过的知情同意书副本。

5.我已得知参与本研究的医生、相关主管部门的负责人以及解放军总医院医学伦理委员会 均有权审阅研究记录和病例资料，我同意上述方面的人员直接得到我的研究记录，并了解 上述信息将得到保密处理。

6. 经过充分考虑后，我自愿参加本临床研究。

受试者签名： 日期：

姓名（正楷）： 受试者联系电话：

研究者声明：

我确认已向患者解释了本研究的详细情况，特别是参加本研究可能产生的风险和受 益，并给受试者一份双方签署过姓名和日期的知情同意书副本。

研究者：吕医生 研究者联系电话：13501149301
